# Evaluation of Knee Position Sense in Children with Motor Disabilities and Children with Typical Development: A Cross-Sectional Study

**DOI:** 10.3390/children10061056

**Published:** 2023-06-13

**Authors:** Åsa Bartonek, Marie Eriksson, Annika Ericson, Mikael Reimeringer, Cecilia Lidbeck

**Affiliations:** 1 Division of Paediatric Neurology, Department of Women’s and Children’s Health, Karolinska Institutet, 17176 Stockholm, Sweden; marie.eriksson@ki.se (M.E.); annika.ericson@ki.se (A.E.); mikael.reimeringer@ki.se (M.R.); cecilia.lidbeck@ki.se (C.L.); 2 Motor Control Laboratory QA:27, Astrid Lindgren’s Children’s Hospital, Karolinska University Hospital, Karolinska Vägen 37A, 17176 Stockholm, Sweden

**Keywords:** arthrogryposis, cerebral palsy, joint position sense, myelomeningocele, proprioception

## Abstract

Background: In children with motor disabilities, knee position during walking is often of concern in rehabilitation. This study aimed to investigate knee joint position sense. Thirty-seven children with Cerebral Palsy (CP), 21 with Myelomeningocele (MMC), 19 with Arthrogryposis (AMC), and 42 TD children participated in the study. Knee joint position sense, i.e., the difference between the criterion angle and the reproduced angle (JPS-error), was assessed in sitting while 3D motion capture was recorded at flexed knee 70 (Knee70), 45 (Knee45), and 20 (Knee20) degrees, and after three seconds at maintained criterion angle (CAM) and maintained reproduced angle (RAM). No differences were found between the groups in JPS-error, CAM, and RAM. At Knee70, CAM differed between the right and left legs in the TD group (*p* = 0.014) and RAM in the MMC group (*p* = 0.021). In the CP group, CAM was greater than RAM at Knee70 in the left leg (*p* = 0.002), at Knee45 in both legs (*p* = 0.004, *p* = 0.025), and at Knee20 in the right leg (*p* = 0.038). Difficulties in maintaining the knee position at CAM in the CP group sheds light on the need for complementary judgments of limb proprioception in space to explore the potential influence on knee position during walking.

## 1. Introduction

In children with motor disabilities, altered knee position during walking is often of concern in rehabilitation. The cause of the observed difficulties varies with respect to the nature of the motor disability. In some children, knee flexion during walking is encountered, whereas, in others, knee hyperextension is observed.

Children with bilateral spastic Cerebral Palsy (CP), Myelomeningocele (MMC), and Arthrogryposis Multiplex Congenita (AMC) are often referred to gait laboratories for evaluation of walking function.

CP is a descriptive diagnosis including a group of movement and posture disorders attributed to an injury in the developing brain. Apart from motor disorders, disturbances with sensation, perception, cognition, communication, behavior, and epilepsy are common [1]. The current overall CP birth prevalence for high-income countries is 1.6 per 1000 live births [2]. The motor impairments, with reduced motor repertoire, hypertonia, and progressive muscle changes related to neuronal-, nutritional- and/or mechanical factors, may lead to musculoskeletal deformities [3]. In ambulating children with spastic cerebral palsy, crouch gait, characterized by increased knee flexion throughout stance, is a common gait pattern that is very tiring to sustain and, in some cases, accompanied by rushing forward [3]. Dropping into increasing ankle dorsiflexion and knee and hip flexion soon after rising to stand has been interpreted as an exhaustibility of the support reaction of the legs in children with spastic CP [4].

MMC is one of the most common congenital malformations, occurring in approximately 1 per 1000 births worldwide. MMC is a birth defect in which the vertebral column is open and characterized by failure of the lumbosacral spinal neural tube to close during embryonic development, resulting in a neurological deficit involving both motor and sensory functions. Urinary and fecal incontinence, hindbrain herniation, and associated hydrocephalus, as well as orthopedic abnormalities, are frequently observed [5]. The neurological level of the lesion is the most determining for walking ability; additional influencing factors such as spasticity, contractures, and balance disturbances have been found related to ambulatory function [6]. At a low neurological level, ambulating children with MMC typically walk with increased knee flexion but cannot stand still with subsequent need for external stabilization of the ankle [7].

AMC is a term representing a heterogenous condition, with the descriptive definition of a baby born with congenital contractures in more than one body area. Over 400 specific conditions that can lead to a baby being born with multiple contractures have been identified, and a responsible gene has been found in more than 150 of these conditions [8]. All forms of AMC are associated with decreased fetal movement (fetal akinesia), with a direct relationship between the duration of decreased movements and the severity of the contractures at birth. The contractures are usually non-progressive and improve over time with early physiotherapy and appropriate orthopedic and orthotic care. Although AMC has been considered a rare condition, it occurs between one in 3000 and one in 5000 live births [9]. In children with AMC, both knee hyperextension and increased knee flexion while walking have been observed [10]. Assessment of motor performance and gait in persons with motor disabilities is commonly based on a comprehensive physical examination, including measurements of joint range of motion, muscle strength, and spasticity. Due to the importance of proprioception in motor planning and motor coordination, it has been suggested that clinicians assess proprioception when assessing motor skills [11]. The proprioceptive senses, including the sense of position and movement of limbs and trunk, the sense of effort, the sense of force, and the sense of heaviness, are generated as a result of an individual’s own actions. Receptors involved in proprioception are located in the skin, muscles, and joints and provide information about limb position and movement to determine the location of the limbs in space [12]. For motor behaviors such as locomotion, afferent input related to load and hip-joint position probably has an important role in the contribution to the activation pattern of the leg muscles [13]. In more dynamic balance-demanding activities, such as walking on various surfaces, both proprioceptive and vestibular capabilities are required [11]. It is important to identify the causes of impaired proprioception since it plays a crucial role in the postural control system, fixing the orientation and position of the segments that serve as a reference frame for perception and action with respect to the external world [14]. Joint proprioception can be measured through indirect methods, such as parents’ reports or clinician observation checklists that can be used as screening tools to identify persons in need of further proprioceptive testing. A direct assessment of proprioception has been suggested to provide the most accurate information about the proprioceptive sense [11]. When examining the reliability of knee joint position sense measurements in healthy adults, sitting was preferred to the prone position for calculation of the absolute error between the target and estimated position in the ipsilateral leg [15].

It has been assumed that in children with bilateral CP, the proprioceptive deficit may contribute to walking difficulties, as shown by the need for compensatory visual control of the feet [16,17] and by a preference for floor mobility even in children who are able to walk [18]. In children with spastic CP, proprioception error in the hip joint was related to postural sway and walking speed [19,20]. In individuals with CP, joint position sense errors in limbs with exaggerated reflexes were comparable with those of typically developing participants, whereas they were not in limbs without hyperreflexia, suggesting that the increased activation of muscle spindles may provide some compensatory effect on joint position sense [21]. In various forms of AMC, there are reports of deviant muscle development and function, likely due to the loss of specific proteins in afferent neurons, leading to disturbed proprioception [22] with an unstable wide-based gait and coordination defects [23]. Since in MMC, both motor and sensory deficits are present [6], it seems plausible that gait pattern [24] is influenced by sensory dysfunction as well. In infants with MMC at a lumbosacral level, investigations have demonstrated that at neurological levels that affect major locomotor muscles, the infants find ways to respond and adapt their motor output to changes in sensory input after treadmill stepping [25].

In children with CP, musculoskeletal deformities in the lower limbs are commonly addressed by both skeletal and soft-tissue surgery [26]. In the orthopedic field of MMC, common procedures are the treatment of knee flexion contractures in ambulatory patients [27]. In the management of lower limb deformities in children with AMC, orthopedic surgery is performed to improve functional outcomes with early management in intensive physiotherapy and bracing [28]. In typically developing children and adolescents, the threshold to detect passive motion was increased after a knee ligament injury contributing to proprioceptive deficits [29]. In view of this, it is assumed that children with motor disabilities with associated joint deformities [10], muscle paresis [5], or disturbed muscle morphology [30] may have reduced proprioception in the knee. Our hypothesis was that children with central nervous system involvement, such as CP, and children with predominantly joint deformities (AMC) or peripheral lesions (MMC) would present with reduced knee proprioception compared to children with Typical Development (TD).

## 2. Methods

### 2.1. Participants

Children with motor disabilities who are commonly referred to the assessment of gait and motor functioning in the neuropediatric department of Karolinska University Hospital, Stockholm, Sweden, were invited to participate in a cross-sectional study between January 2019 and December 2020. Seventy-seven participants between the ages of 5 and 18 years were grouped according to their diagnosis: 37 with bilateral spastic CP, 21 with MMC, and 19 with AMC. All participants had an ambulatory function; however, two participants with CP walked only for transfer in a closed environment, and one participant with MMC had ceased walking as a teenager. Ambulation according to the various diagnosis was documented with the Gross Motor Function Classification System (GMFCS E&R) [31] in children with CP, and according to Hoffer in the MMC [32] and AMC [33] groups. Performed orthopedic surgery was documented in the medical chart. Participants had to meet the following criteria: knee extension muscle strength “good” (Grade 4) with the ability to withstand considerable manual resistance when being asked to hold the leg not allowing the examiner to “break” the hold or “normal” (Grade 5) with ability to maintain end-point range against maximal resistance [34]; passive knee motion available to perform the test; and no orthopedic surgery in the last year. In addition, the ability to understand verbal instructions necessary to accomplish the test was required. Forty-two individuals with TD constituted a control group. The TD children were recruited among siblings of patients, through advertisements at the hospital, and among acquaintances of the researchers.

The study was approved by the Regional Ethical Review Board in Stockholm, code nr 2017/953-31/2. Before taking part in the study, written informed consent was given by the caregivers, and a verbal agreement was confirmed by the children.

### 2.2. Knee Joint Position Sense

**Figure 1:**
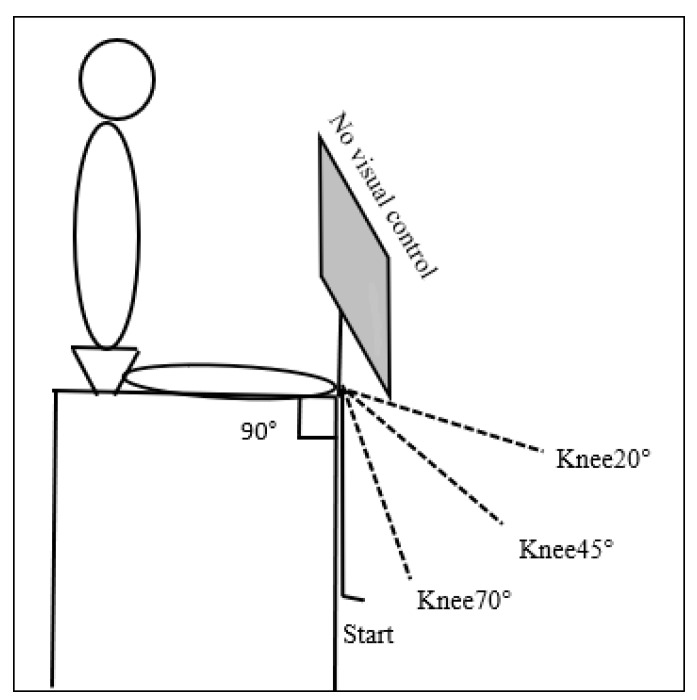
Measurements of knee joint position sense performed at 70, 45, and 20 degrees of knee flexion.

**Figure 2:**
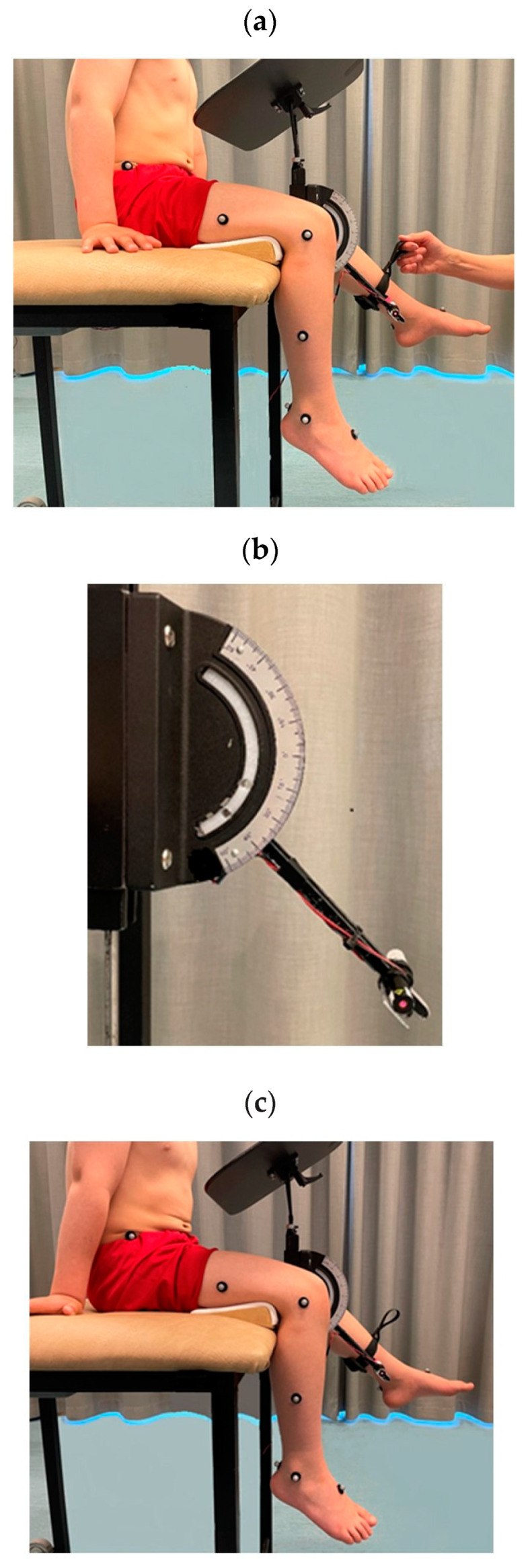
Equipment and procedures for measuring knee joint position sense. The participant sat on a stool with a tray table hindering visual control while (**a**) the leg was passively elevated by the examiner to the criterion angle, (**b**) guided by a red laser pointer attached to a goniometer, and (**c**) the child was instructed to raise the leg actively to reproduce the criterion angle.

Knee joint position sense was measured in a sitting position on a customized stool with a 60 × 60 cm area seat and a height of 70 cm. A wedge was placed under the distal part of the thigh to ensure a hip flexion angle of 90 degrees and to adjust for a knee flexion angle of 90 degrees. The legs hung freely to eliminate any external cues. The participants were examined barefoot and dressed in shorts. Examinations were performed in both legs at three knee flexion angles, namely 70 (Knee70), 45 (Knee45), and 20 (Knee20) degrees [35]. A height-adjustable tray table was arranged in front of the participant to prevent visual control of leg movements during testing (Figure 1). By gripping a molded strap with a soft lining to minimize cutaneous sensation, placed distally at one-third of the participant’s shank length, the examiner passively elevated the participant’s leg to a Criterion Angle (CA) (Figure 2a). To set the CA, the examiner was guided by a red laser pointer which was placed on the stool attached to a goniometer (Figure 2b). Next, the participant was asked to actively maintain and memorize the CA position for three seconds. After a resting period of approximately 5 s, the participant was asked to reproduce the leg position at the CA by actively lifting the leg to the perceived position, giving the Reproduced Angle (RA), and maintaining that position for 3 s before returning their leg to its resting position (Figure 2c). The trials started with a knee flexion angle of 70 degrees, followed by 45 and then 20 degrees. The left and right legs were tested in randomized order. Prior to the test, thorough explanations were given to the participant, and familiarization trials were undertaken to ensure the participant’s understanding of the test procedure. There were two trials for each angle, with a short break of approximately ten seconds between trials. Some participants had difficulty actively maintaining the knee position for three seconds; additional measurements were therefore made to assess the ability to maintain leg positions at the CA (Criterion Angle Maintained, CAM) and at the RA (Reproduced Angle Maintained, RAM).

The JPS test was performed at the Motion Analysis Laboratory at Karolinska University Hospital on one occasion. Four examiners, specialized in each of the three patient categories, were involved in data collection.

### 2.3. Motion Analysis

The knee angles were recorded with 3D motion analysis using a 12-camera system (Vicon MX40^®^ Oxford, UK) with a sampling rate of 100 Hz. The stool was placed in the middle of the laboratory room so that all markers (attached in sitting position) were visible during data capture, with the participant’s hands placed on the sides of the stool to avoid obscuring the thigh markers. A kinematic lower body model consisting of 16 reflective markers, of which eight were on each side, were attached on the anterior superior iliac spine, posterior superior iliac spine, thigh, femur condyle, shank, lateral malleolus, heel, and forefoot, according to the Newington model [36]. Video recordings were made simultaneously.

### 2.4. Data Analysis

Vicon Nexus was used to process the trials. Data were missing in the TD group: in one left trial at Knee70; in the CP group: in 11 left and right trials at Knee20; in the MMC group: in one left trial at Knee45 and three left and two right trials at Knee20; and in the AMC group: two left and four right trials at Knee70, one right trial at Knee45, and three left and right trials at Knee20.

**Figure 3:**
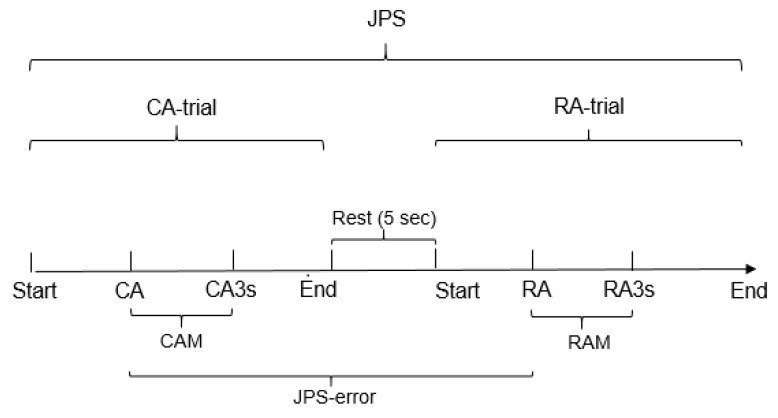
Events during the measurement of knee position sense (JPS). CA = Criterion Angle, RA = Reproduced Angle, CAM = CA − angle at 3 s (CA-3s), RAM = RA − angle at 3 s (RA-3s), JPS-error = CA − RA.

The ability to sense knee joint position was assessed using the absolute difference in degrees between the CA and RA (JPS-error) [37]. The knee position angle after three seconds relative to the CA (CA-3s) and the knee position angle after 3 s relative to the RA (RA-3s) provided an assessment of the ability to maintain the knee position by taking the difference in degrees between the CA and CA-3s (CAM) and between the RA and RA-3s (RAM) (Figure 3).

### 2.5. Statistics

For each leg and each of the measured angles, the trial with the smallest difference between the CA and RA (that is, the best-performing trial) was chosen for analysis. Descriptive data are presented in terms of medians and min-max. Nonparametric methods were used due to the absence of normally distributed data. A Kruskal–Wallis test with a post-hoc comparison using the Mann–Whitney U test was used to analyze possible differences in age, weight, and height and performed orthopedic surgery between the TD, CP, MMC, and AMC groups. A Wilcoxon test was used to analyze possible differences between the left and right legs in JPS-error, CAM, and RAM and differences between the CAM and RAM according to each of the disability groups. To explore possible differences in JPS-error, CAM, and RAM between groups, a Kruskal–Wallis test was applied, and differences in JPS-error with respect to ambulatory function within each of the CP, MMC, and AMC groups. Statistics were computed using commercially available software (SPSS, version 26) at a significance level of 0.05.

## 3. Results

Participant characteristics, orthopedic surgery, and ambulatory function in participants with TD, CP, MMC, and AMC are shown in Table 1. The median ages for the CP and MMC groups were higher than the TD group (*p* = 0.008 and *p* = 0.016, respectively). There were no differences between the TD, CP, MMC, and AMC groups with respect to height, weight, or gender. Orthopedic surgery had been performed significantly more in participants in the MMC and AMC groups than in the CP group, in the ankle (*p* = 0.005 and <0.001 respectively) and in the knee (<0.001 and knee 0.006 respectively), and more in the hip in MMC than in the CP group (*p* = 0.037).

### 3.1. JPS-Error

The JPS error did not differ significantly in any leg between the TD, CP, MMC, and AMC groups at Knee70, Knee45, or Knee20 (Table 2). There were no differences in the JPS error between the left and right legs. No differences were found in JPS-error at Knee70, Knee45, or Knee20 with respect to the GMFCS levels in the CP group or to ambulation groups in the MMC and AMC groups.

### 3.2. CAM

There were no significant differences in CAM between the TD, CP, MMC, and AMC groups at Knee70, Knee45, or Knee20 (Table 2). With respect to left and right legs, CAM differed at Knee70 in the TD group (*n* = 42), with median [min, max] degrees of 3 [0, 12.1] versus 2 [0, 11.2], *p* = 0.014.

### 3.3. RAM

There were no statistically significant differences in maintaining any leg at RAM between the TD, CP, MMC, and AMC groups at Knee70, Knee45, or Knee20 (Table 2). With respect to left and right legs, RAM differed at Knee70 in the MMC group (*n* = 21), with median [min, max] degrees of 1.0 [0, 10.2] versus 2.5 [0.3, 6.9], *p* = 0.021.

**Figure 4:**
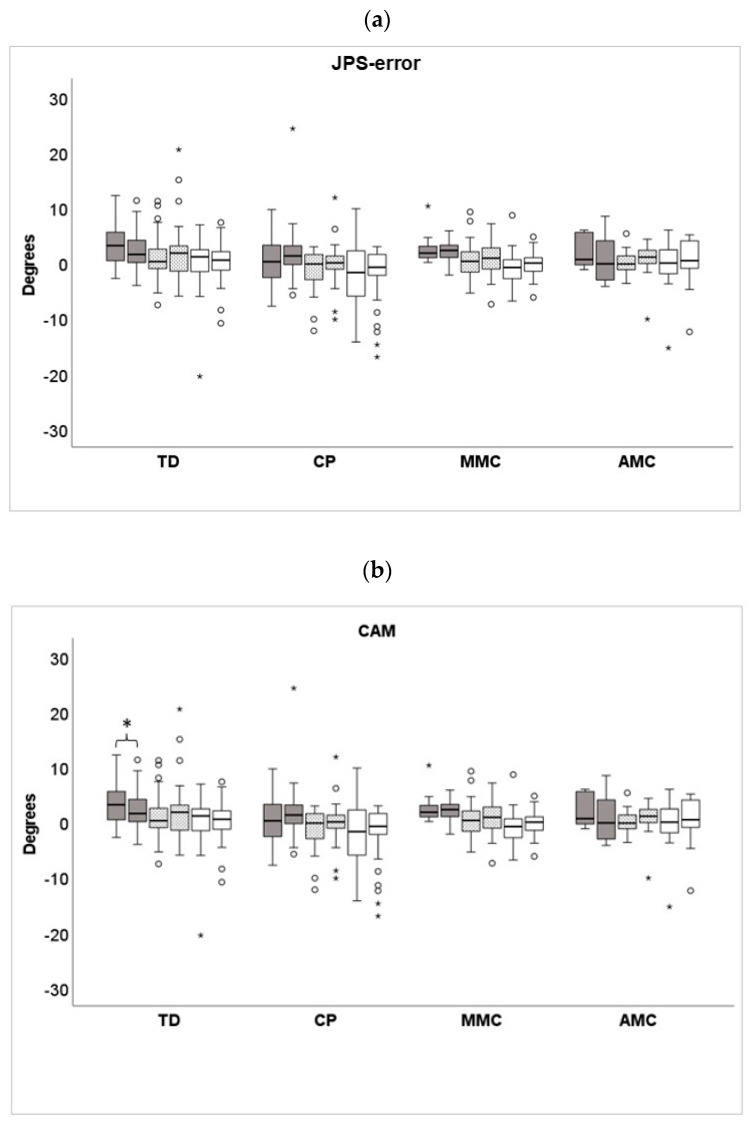

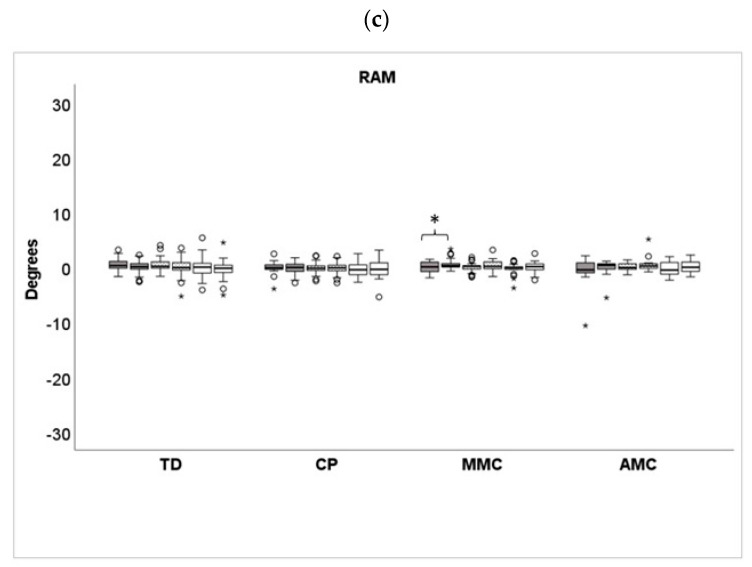
(**a**–**c**) Relative errors as over-estimation (+) of the leg movement and under-estimation (−) of the leg movement with respect to the target position of joint position sense error (JPS-error) defined as the difference between the Criterion Angle (CA) and Reproduced Angle (RA), CAM (difference between the CA versus CA maintained for 3 s) and RAM (difference between the RA versus RA maintained for 3 s), presented as median degrees and 25th–75th percentiles in groups of children with Typical Development (TD), Bilateral Spastic Cerebral Palsy (CP), Arthrogryposis Multiplex Congenita (AMC) and Myelomeningocele (MMC). Left and right values for JPS-error, CAM, and RAM at the tested angles of Knee70 degrees are represented by grey bars, Knee45 degrees by dotted bars, and Knee20 degrees by white bars. * indicate significant differences between the left and right legs.

Figure 4a–c illustrates relative errors of over- and under-estimating the target position at the JPS-error, CAM, and RAM [37].

### 3.4. CAM vs. RAM

CAM was greater than at RAM in the CP group in the left leg Knee70 with median [min, max] degrees of 1.04 [0, 6.37] versus 0.54 [0.35, 3.95], *p* = 0.002, in the left leg Knee45 median [min, max] degrees 1.34 [0.01, 20.14] versus 0.5 [0.47, 3.3], *p* = 0.004), in the right leg Knee45 median [min, max] degrees 1.17 [0.48, 11.74] versus 0.66 [0.04, 3.57] *p* = 0.025, and in the right leg Knee20 median [min, max] degrees 1.26 [0.04, 18.19] versus 1.05 [0.11, 5.45] *p* = 0.038.

## 4. Discussion

To our knowledge, this is the first study examining knee joint position sense involving children with various motor disabilities who are commonly referred for assessments of gait and or motor functioning at a neuropediatric department. Despite the often observed altered knee position during walking in these children, our hypothesis that knee joint position sense was reduced in children with CP, AMC, and MMC compared to TD children could not be confirmed. When addressing gait in children with motor disabilities, it is common to determine a more or less dominant leg. Since the investigation was performed in the sitting position with the inclusion criteria being good to normal muscle strength [34] of the knee extensors, we did not consider a choice of a more involved side to be of importance in this study. Accordingly, when calculating the differences between the sides, no differences in JPS error were found, but they were present at Knee70 in CAM in the TD group and in RAM in the MMC group, values that were not considered clinically relevant. In the CP group, however, the highest JPS-error values with respect to the other disability groups were found but did not reach a significant level. Conversely, orthopedic surgery in the lower limbs, even in the knee, had been performed significantly less among participants in the CP group than in the MMC and AMC groups. This finding, therefore, does not further elucidate the possible influence of orthopedic surgery on the JPS results. Nor when discriminating for functional ambulatory level, differences in JPS error with respect to each of the diagnosis groups were found. Furthermore, in children with MMC with a paresis at a low neurological level, knee flexed position in an upright position is plausible due to insufficient calf muscle innervation. However, even though motor paresis is present in the biceps femoris muscle [8] at a low neurological level, this did not seem to have any impact on knee joint position sense in the unloaded sitting position. Nor did the often-seen knee joint deformities in AMC [10] seem to play a determinant role in sensing knee position. Among the children with MMC, in one child with an asymmetric neurological lesion level, one leg was excluded due to insufficient quadriceps muscle strength, requiring a locked knee-ankle-foot orthosis when walking. All other children with MMC requiring orthoses were able to walk with non-restricted knee joints indicating at least good (grade 4) quadriceps strength [34]. There is important evidence that muscle spindles, being the principal kinaesthetic sensors, play a major role in kinaesthesia [13]. Since all children included in this study were able to activate their knee extensor muscles against manual resistance [34], one could speculate that muscle activation of the innervated knee extensors contributed sufficiently to the ability to sense the knee position in sitting, showing similar results in all groups. However, when grading muscle strength, it is challenging to minimize the subjective element by the examiner when performing the measurement [34].

The reason for assessing CAM and RAM derived from the experienced effort by some children to maintain the leg at the criterion angle set by the examiner versus when maintained at the self-initiated movement at the reproduced angle. The results confirm that there were discrepancies between the CAM and RAM in the CP group presenting with significantly larger CAM at four of the six tested angles, ranging between maximum values of approximately six degrees at Knee70 to eight to 17 degrees at Knee45 and 13 at Knee20, respectively. The difficulty in the CP group to maintain the knee joint extended for three seconds after a not self-initiated movement raises further questions about movement perception and motor planning in children with CP. However, a limitation of the study is that CAM and RAM have not been reported previously when assessing JPS and must be validated in larger groups. A further limitation of the study is the reduced sample size of children in the CP group that could perform the twenty-degree knee flexion trial (26 out of 37) due to contractures and/ or difficulties in selectively performing the task. Another weakness of the study is that in some (15 out of 19) of the children with AMC, the seventy-degree angle test could not be performed because of insufficient passive knee flexion. A limitation of the study is also the relatively small number of participants which precludes the generalization of our results.

According to Olsson et al. [16], it may be easiest for a participant to reproduce the position of the leg movement in sitting at a 70-degree knee angle since it is close to the start position of the 90-degree angle. In our study, with similar results at all tested angles, no variations with respect to the knee flexion angles of 70, 45, or 20 degrees could be confirmed. In the study by Olsson et al. [16], including healthy adults, participants were examined during five trials in each of the three test positions. The same cognitive attention span required could not be expected in children, particularly not within the CP group, since attentional disturbances may be present by definition [3]. In our study, the decision to perform two trials for each angle was based on the experiences from our pilot study, indicating short attention spans in some of the children. Experiences within the pilot study also led to the decision to minimize the time spent sitting on the equipment stool during preparation. The markers for kinematic data were therefore applied to the child’s body in advance while the child was seated on a regular chair, allowing adjustments of marker placement to be made as close as possible to the start of the proprioception trials. The markers were applied by examiners who were trained in the gait laboratory, and the same technician performed all kinematic data collection. Since full attention to the interaction between examiner and child could not always be maintained throughout the trials, some measurements should be interpreted with caution.

Impaired joint position sense of lower extremities measured isolated in ankles and knees has been reported to correlate with balance function in children with developmental coordination disorder [38]. Some authors, however, recommend estimating body position during standing in children to provide information on joint positions of the ankle and from the loading of foot mechanoreceptors [39]. However, in many children with motor disabilities, muscle weakness, spasticity, or exhaustibility of the support reaction lead to insufficient stabilization of ankle joints, impeding taking valid measurements in the standing position. Providing an ankle-foot orthosis would ensure a stable ankle joint, but the impact on other body segments and their contribution to postural control would remain unclear. Although, since in the present study, knee JPS per se, as investigated in the sitting position, cannot be considered to primarily contribute to difficulties in controlling the knee joint during walking, studies on isolated ankle joint position sense may add further information. For such a study, however, factors of muscle strength, movement selection, and sensation should be taken into consideration.

## 5. Conclusions

By investigating knee joint position sense with 3D-motion analysis in a non-weight-bearing position in children with sufficient knee extension muscle strength, we found no significant differences in JPS-error, CAM, or RAM between any of the groups with motor disabilities compared to children with TD, neither with respect to age nor ambulatory level. Lately, suggestions have been made to incorporate proprioceptive low-level tests detecting such as joint matching movements and proprioceptive high-level judgments indicating the location of limbs in space performed in a different frame of reference [40]. Most recently, proprioception was defined as the sense of where one’s limbs are in space in a study identifying the role of somatosensory neurons for musculoskeletal development in children with distal AMC [41]. Accordingly, the discrepancy found between CAM and RAM in the CP group in the present study sheds light on the need for complementary assessments using joint position matching and detecting leg position in space [40]. This will benefit further exploration of the role of proprioception and its potential influence on knee position during walking with respect to various motor disabilities.

## Figures and Tables

**Table 1:** Participant characteristics, orthopaedic knee surgery, and ambulatory function in participants with typical development (TD), bilateral spastic cerebral palsy (CP), myelomeningocele (MMC), and arthrogryposis multiplex congenita (AMC).

	TD (*n* = 42)	CP (*n* = 37)	MMC (*n* = 21)	AMC (*n* = 19)	*p*-Value
Age; median [min, max] years	10.9 (6.7, 18)	14.4 (6.4, 18)	15.1 (8.7, 18)	13.2 (5.8, 17.4)	0.019 *
Height; median [min, max] cm	152.5 (118, 189.8)	154 (113.1, 178)	149 (107.5, 176)	150 (110, 179)	0.302
Weight; median [min, max] kg	39.5 (22.1, 84.5)	49.1 (21, 73)	49.0 (26, 9)	41.1 (18.2, 71.6)	0.208
Gender f/m	22/20	12/25	11/10	9/10	0.290
Orthopedic surgery, uni-and bilateral					
- Total hip, knee, ankle joints nr participants (%)	-	13 (35)	16 (76)	18 (95)	<0.001
- Ankle		12 (32)	15 (71)	16 (84)	<0.001
- Knee		5 (14)	13 (62)	9 (47)	<0.001
- Hip		4 (11)	7 (33)	5 (26)	0.103
Ambulatory function		GMFCS I: 12, II: 10, III:15	Ca: 7 Ha: 7 N-f: 6 N-a: 1	Ca: 16 Ha: 3	

* indicates *p* < 0.05. GMFCS = Gross Motor Function Classification Scale, Ca = Community Ambulation, Ha = Household Ambulation, N-f = Non-functional ambulation, N-a = Non-ambulation.

**Table 2:** Joint-position sense as the difference between the Criterion Angle (CA) and Reproduced Angle (RA) (JPS error), CA with respect to CA maintained for 3 s (CAM), and RA with respect to RA maintained for 3 s (RAM) presented as median absolute errors in participants With Typical Development (TD), Bilateral Spastic Cerebral Palsy (CP), Myelomeningocele (MMC), and Arthrogryposis Multiplex Congenita (AMC). “*n*” indicates performed trials within each group.

Angle (Degrees)	*n* (Legs)	TD (*n* = 42)	*n* (Legs)	CP (*n* = 37)	*n* (Legs)	MMC (*n* = 21)	*n* (Legs)	AMC (*n* = 19)	*p*-Value
Median [min, max]						JPS error			
Left 70	42	3 (0, 12.1)	37	1.7 (0, 9.6)	21	1.8 (0, 10.2)	17	1.7 (0.1, 5.9)	0.517
Right 70	42	2 (0, 11.2)	37	1.6 (0, 24.2)	21	2.5 (0.8, 6.9)	15	3.7 (0.7, 10.9)	0.073
Left 45	42	1.8 (0, 11.1)	37	2.3 (0, 14.9)	20	2.8 (0.1, 9.2)	19	1.3 (0.2, 5.2)	0.201
Right 45	42	2.4 (0.1, 20.4)	37	1.6 (0, 11.7)	21	2 (0.2, 7.5)	18	2 (0.2, 10.2)	0.460
Left 20	41	2.2 (0.1, 20.6)	26	3.6 (0.3, 14.3)	18	2.4 (0, 8.6)	16	2.3 (0.2, 15.4)	0.096
Right 20	42	2.49 (0.2, 10.6)	26	1.87 (0.2, 17.1)	19	1.53 (0.1, 6.3)	16	2.24 (0.1, 12.5)	0.383
	CAM								
Left 70	42	0.53 (0, 2.9)	37	1.03 (0, 6.4)	21	0.6 (0, 3.6)	17	0.5 (0, 3.4)	0.265
Right 70	42	0.4 (0, 3.5)	37	0.8 (0, 5.2)	21	0.5 (0, 5.6)	15	0.4 (0, 4.1)	0.432
Left 45	42	0.5 (0,4.4)	37	1.3 (0, 20.1)	20	0.8 (0.1, 3.5)	19	0.4 (0.1, 3.5)	0.056
Right 45	42	0.6 (0, 3.5)	37	1.2 (0, 11.7)	21	0.8 (0, 8.6)	18	0.8 (0.1, 2.6)	0.062
Left 20	41	0.74 (0, 5)	26	2.01 (0, 16.6)	18	0.85 (0, 4.3)	16	1.09 (0, 7.5)	0.133
Right 20	42	0.75 (0, 4.2)	26	1.26 (0, 18.2)	19	0.60 (0, 5.8)	16	0.96 (0, 6.3)	0.059
		RAM							
Left 70	42	0.7 (0, 3.2)	37	0.5 (0, 0.2)	21	0.8 (0, 2)	17	1 (0, 10.7)	0.449
Right 70	42	0.5 (0.4, 2.7)	37	0.6 (0, 7.4)	21	0.4 (0, 3.3)	15	0.6 (0,1, 5.6)	0.409
Left 45	42	0.5 (0, 4)	37	0.5 (0, 3.3)	20	0.4 (0, 2)	19	0.3 (0.3, 2)	0.705
Right 45	42	0.7 (0, 5.3)	37	0.7 (0, 3.6)	21	0.8 (0, 3.1)	18	0.5 (0.1, 5.1)	0.664
Left 20	41	0.8 (0, 5.3)	26	1 (0.1, 2.8)	18	0.5 (0, 3.9)	16	0.7 (0.2, 2.4)	0.445
Right 20	42	0.7 (0.1, 5.1)	26	1.1 (0.1, 5.4)	19	0.6 (0.1, 2.5)	16	1 (0, 2.2)	0.331

## Data Availability

The datasets generated during and analyzed during the current study are available from the corresponding author upon reasonable request.

## Abbreviations

TD	Typical Development
MMC	Myelomeningocele
AMC	Arthrogryposis Multiplex Congenita
CP	Cerebral Palsy
Knee70	70-degree knee flexion angle
Knee45	45-degree knee flexion angle
Knee20	20-degree knee flexion angle
JPS	Joint Position Sense
CA	Criterion Angle
RA	Reproduced Angle
CA-3s	Maintaining the CA for 3 s
RA-3s	Maintaining the RA for 3 s
CAM	Difference between CA and CA after maintaining the position for 3 s
RAM	Difference between RA and RA after maintaining the position for 3 s
